# Esophageal Achalasia in Parkinson's Disease: Diagnosis and Management of a Rare Case

**DOI:** 10.37825/2239-9747.1052

**Published:** 2024-07-08

**Authors:** Alexandros Ioannou, Francesco Torresan

**Affiliations:** a Gastroenterology Department, Alexandra General Hospital, Athens, Greece; b Gastroenterology Unit, Department of Medical and Surgical Sciences, University of Bologna, Bologna, Italy

**Keywords:** Esophageal achalasia, Parkinson disease, High resolution manometry

## Abstract

Autonomic dysfunction in Parkinson's disease involving the gastrointestinal track due to the presence of inclusions of a-synuclein in the vagus nerve and in the Meissner and Auerbach plexus of the enteric nervus system. Esophageal achalasia characterized by a degenerative loss of the inhibitory neurons in the myenteric plexus that causes a failure of the lower esophageal sphincter to relax leading to dysphagia, regurgitation, retrosternal pain and weight loss. We report a case of a Parkinson disease patient presented to our department due to upper gastrointestinal symptoms, diagnosed with esophageal achalasia by high resolution esophageal manometry and successfully treated with pneumatic dilatation.

## 1. Introduction

Parkinson's disease (PD) is the most common neurodegenerative condition affecting almost 1% of the aged population [1]. The two pathological hallmarks of PD are a progressive degeneration of the dopamine-containing neurons in the *substantia nigra pars compacta* combined with intraneuronal aggregates of eosinophilic inclusions, mainly phosphorylated a-synuclein [2,3]. Although PD is regarded as a movement disorder, non-motor manifestations, such as autonomic dysfunctions, particularly those involving the gastrointestinal (GI) tract (dysphagia and constipation), are increasingly recognized [4]. In particular, the enteric nervous system (ENS) is a prime target of the pathological process of PD. Indeed, autopsies of parkinsonian patients reveal the widespread presence of a-synuclein aggregates in myenteric and submucosal neurons throughout the GI tract [5].

Achalasia is an esophageal motility disorder with a prevalence of ~1: 10 000. Dysphagia consists of the main clinical manifestation followed by regurgitation, retrosternal pain and weight loss. A degenerative loss of the inhibitory neurons of the myenteric plexus causing absence of esophageal peristalsis and failure of relaxation of the lower esophageal sphincter represent main pathogenetic basis [6,7]. However, some studies highlighted that the location of denervation could be preganglionic and that postganglionic nerves could be intacted, suggesting a central origin for primary achalasia [8]. Epidemiologic studies suggest that achalasia appears to represent the clinical endpoint of several pathways. Not only aging but also different neurologic diseases may contribute to a loss in control of esophageal motility [9].

We report the case of a 63-year-old woman affected by Parkinson's disease that was referred to our department due to dysphagia, regurgitation, retrosternal pain, and weight loss of 15 kg in the last 2 months and diagnosticated with esophageal achalasia.

## 2. Case report

A 63-year-old female patient, diagnosed with PD four years ago, presenting mild rigidity and resting tremor successfully treated with levodopa/benserazide. The patient was also affected by non-motor PD symptoms, namely depression and mild constipation.

**Fig. 1:**
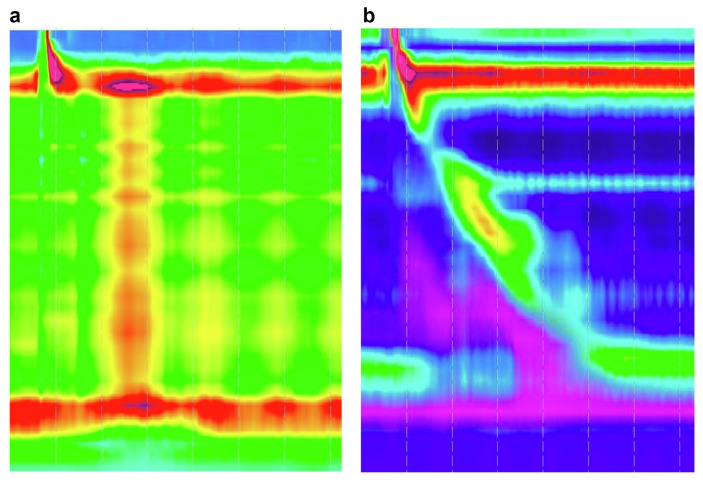
High resolution esophageal manometry (a) at the diagnosis and (b) after the second pneumatic dilatation.

She was referred to our clinic, presented dysphagia in every meal for the last two months, daily regurgitation, daily retrosternal pain and weight loss of 15 kg. (Eckardt score:10). The patient performed: (i) an upper GI endoscopy without any particular finding; (ii) a Video Fluoroscopic Swallowing Exam with a minor difficulty of forming and swallowing the bolus; and (iii) a Rx esophagogram with appearance of barium height of >5 cm at 1 min. Moreover, an examination with a high-resolution esophageal manometry (Fig. 1(a)) that highlighted an esophageal achalasia type II manometric pattern according to Chicago Classification Criteria v4.0 (Integrated Relaxation Pressure (IRP) 34.1 mmHg, 80% panesophageal pressurization). The patient was initially treated with a 30 mm rigiflex balloon dilatation with minor benefit on regurgitation and chest pain, while dysphagia persisted in every meal (Eckardt score: 6). Therefore, a second dilatation with a 35 mm rigiflex balloon was performed. At the follow up visit, after a month of the second dilatation, the patient was in clinical remission without regurgitation, rare episodes of retrostrnal pain, occasional dysphagia and stable weight (Eckardt score: 2) Finally, a second high-resolution manometry was performed (Fig. 1(b)) that revealed improvement of the manometric parameters (IRP 14.3 mmHg).

## 3. Discussion

Dysphagia is a common symptom in PD patients that deteriorate their quality of life. Its etiology is multifactorial and its management challenging [10]. Dysphagia has been usually attributed to oropharyngeal dysfunction detected fluoroscopically and rarely to disordered esophageal motility [11]. Several studies using water-perfused manometry have identified esophageal motility abnormalities in parkinsonian patients [12,13]. Recently, Su et al. evaluated 33 patients, that were affected by PD and experienced dysphagia, by performing high resolution esophageal manometry. According to the Chicago classification criteria a variety of esophageal abnormalities were identified but no patient presented esophageal achalasia [14]. Suttrup et al. studied using high resolution manometry 65 PD patients with dysphagia in different stages of their disease highlighting that esophageal body impairment is frequent in all stages of the disease and possibly reflects a-synucleinopathy in the enteric nervus system [15]. On the other hand, several studies suggest a central origin for primary achalasia involving the dorsal motor nuclei of the vagus and the ambiguous nuclei, the anatomical structures controlling esophageal motility. The causes of these neural lesions remain unknown. Neurotropic virus, such as herpes zoster, selectively attacking neurons of the vagal nucleus were involved [7].

Multiple recent studies suggest an early occurrence of the upper gastrointestinal dysfunction in PD patients [10,16]. Qualman et al. identified Lewy Bodies in degenerating ganglion cells of the esophageal myenteric plexus in achalasia patients [17]. Additionally, Wakabayashi et al. revealed esophageal Lewy Bodies in PD patients with dysphagia, while weren't present in PD patients without dysphagia and controls [5]. These findings suggest that a subset of achalasia and PD patients with dysphagia may have similar mechanisms of neuronal degeneration responsible for the esophageal dysfunction.

Some case reports in the literature suggest a link between PD and achalasia [18,19]. An epidemiological study by Becker et al. revealed that PD was more frequent in parents of achalasia patients than in control subject parents. Case-control studies stratifying for age provides useful data regarding the relation between PD and achalasia given that PD represents an age-related disorder. Notably, Sonnenberg et al. [9] revealed that in elderly achalasia patients, PD was one of the comorbid disorders observed [20]. Currently there are data neither about the high resolution manometric presentation at the diagnosis nor about the results of the treatment with pneumatic dilatation on the manometric tracing of patients with PD and esophageal achalasia.

In our case, a 63-year-old female patient that was diagnosed with PD, four years before, was referred to our department due to upper gastrointestinal symptoms. Performing a high-resolution manometry, the gold standard exam for esophageal motility disorders, highlighted the presence of esophageal achalasia type II according to the Chicago classification criteria v4.0. The patient was treated successfully with pneumatic dilatation.

**Learning points**

Parkinson's disease (PD) frequently involves gastrointestinal (GI) tract (dysphagia, gastroparesis, and constipation) deteriorating patients' quality of life.

Dysphagia in PD is a clinically relevant symptom, usually caused due to Upper Esophageal Sphincter disfunction affecting the oral and pharyngeal phase of swallowing. Esophageal achalasia represents a very rare cause of dysphagia in PD.

High resolution esophageal manometry is the gold standard for the study of esophageal impairment in PD and may therefore define an effective therapeutic treatment in patients with specific esophageal motor disfunction.
